# Effect of time to first ambulation on recurrence after PELD

**DOI:** 10.1186/s13018-020-01608-7

**Published:** 2020-02-27

**Authors:** Fengwei Qin, Zhaofei Zhang, Caixia Zhang, Yonghui Feng, Sineng Zhang

**Affiliations:** 1 Department of Orthopedics, Guangzhou Hospital of Integrated Traditional and Western Medicine, 87 Yingbin Avenue, Huadu District, Guangzhou City, Guangdong Province People’s Republic of China; 2 Department of Surgery, Xinhua Community Health Service Center, No. 8, Nongxin Road, Huadu District, Guangzhou City, Guangdong Province People’s Republic of China

**Keywords:** Lumbar intervertebral disc herniation, PELD, First ambulation time, Recurrence

## Abstract

**Study design:**

Retrospective cohort study.

**Objective:**

To evaluate the effect of time to first ambulation on recurrence after percutaneous endoscopic lumbar discectomy (PELD).

**Methods:**

From July 2017 to August 2018, 90 patients with lumbar intervertebral disc herniation underwent PELD surgery. According to the initial walking time, i.e., the time until the patient could walk after the operation, the operations were divided into three groups: early stage, middle stage, and late stage. The follow-up period was 3 months, and complete follow-up data were obtained. The visual analog scale (VAS) and Oswestry disability index (ODI) scores before the operation, at first ambulation, 1 month after the operation, and 3 months after the operation and the recurrence and incidence rates of high magnetic resonance imaging (MRI) signal in the vertebral endplate area were recorded after the operation.

**Results:**

The success rate was 100% for these 90 cases. The VAS and ODI scores at the first ambulation after the operation significantly improved compared with those before the operation, and the difference was statistically significant. The improvements in the lumbar VAS and ODI scores of the middle- and late-stage groups were better than that of the early-stage group at 1 and 3 months after the operation, and the differences were statistically significant; however, there was no significant difference between the middle- and late-stage groups. The postoperative recurrence rate and rate of high MRI signal in the vertebral endplate area were significantly higher in the early-stage group than in the other two groups, and the difference was statistically significant.

**Conclusion:**

The time to first ambulation after PELD is an important factor affecting the curative effect of the operation. Early ambulation may be one of the factors affecting recurrence after PELD.

Percutaneous endoscopic lumbar discectomy (PELD) has become the most popular minimally invasive technique for the treating of lumbar disc herniation and has been widely used in clinical practice. The advantages of PELD, including the small incision and rapid recovery, are appreciated by many spinal surgeons and patients. With the promotion of the concept of rapid rehabilitation, most spinal surgeons recommend that the patients start walking the day after surgery and even urge the patients to start walking the same day after surgery. However, some doctors recommend that patients stay in bed for 3 or 7 days or longer after surgery. They believe that an adequate time in bed after surgery can reduce complications. The complications and recurrence rates of PELD have attracted much attention, and many of the contributing factors are controversial. Therefore, questions such as whether the time to ambulation after PELD affects the clinical efficacy and recurrence rate and when is the best time to ambulate after PELD will be discussed in this paper, and the specific contents are presented below.

## Data and methods

### Patient selection method

Inclusion criteria include the following: (a) unilateral or bilateral lower extremity pain with or without low back pain; (b) nerve root symptoms such as abnormal sensations in the lower extremities, positive straight leg elevation test, and positive reinforcement test; (c) diagnosis of single-segment lumbar intervertebral disc herniation by computed tomography (CT) and magnetic resonance imaging (MRI) (Fig. 3c–f); (d) failed of standard conservative treatment for 3 months; and (e) PELD operation and follow-up for more than 3 months.

Exclusion criteria include the following: (a) vertebral instability and spinal stenosis; (b) cauda equina syndrome; (c) lumbar spine fracture, tumor, or active infection in the surgical area; and (d) other systemic diseases that do not allow the patient to tolerate surgery.

### General information

Ninety patients were treated with PELD and were divided into three groups according to the time they initially got out of bed: early (group E), middle (group M), and late (group L). There were 15 males and 17 females in group E, with an average age of 40.81 ± 11.82. L3/4, L4/5, and L5/S1 lesions were found in 3, 28, and 1 patients, respectively. In group M, there were 16 males and 14 females, with an average age of 39.20 ± 10.97. The L3/4, L4/5, and L5/S1 lesions were found in 4, 25, and 1 patients, respectively. In group L, there were 15 males and 13 females, with an average age of 37.46 ± 10.83. The L3/4, L4/5, and L5/S1 lesions were found in 3, 23, and 2 patients, respectively. Prior to the operation, all patients underwent anteroposterior and lateral lumbar X-ray, CT, and MRI scans.

### Operative methods

**Fig. 1:**
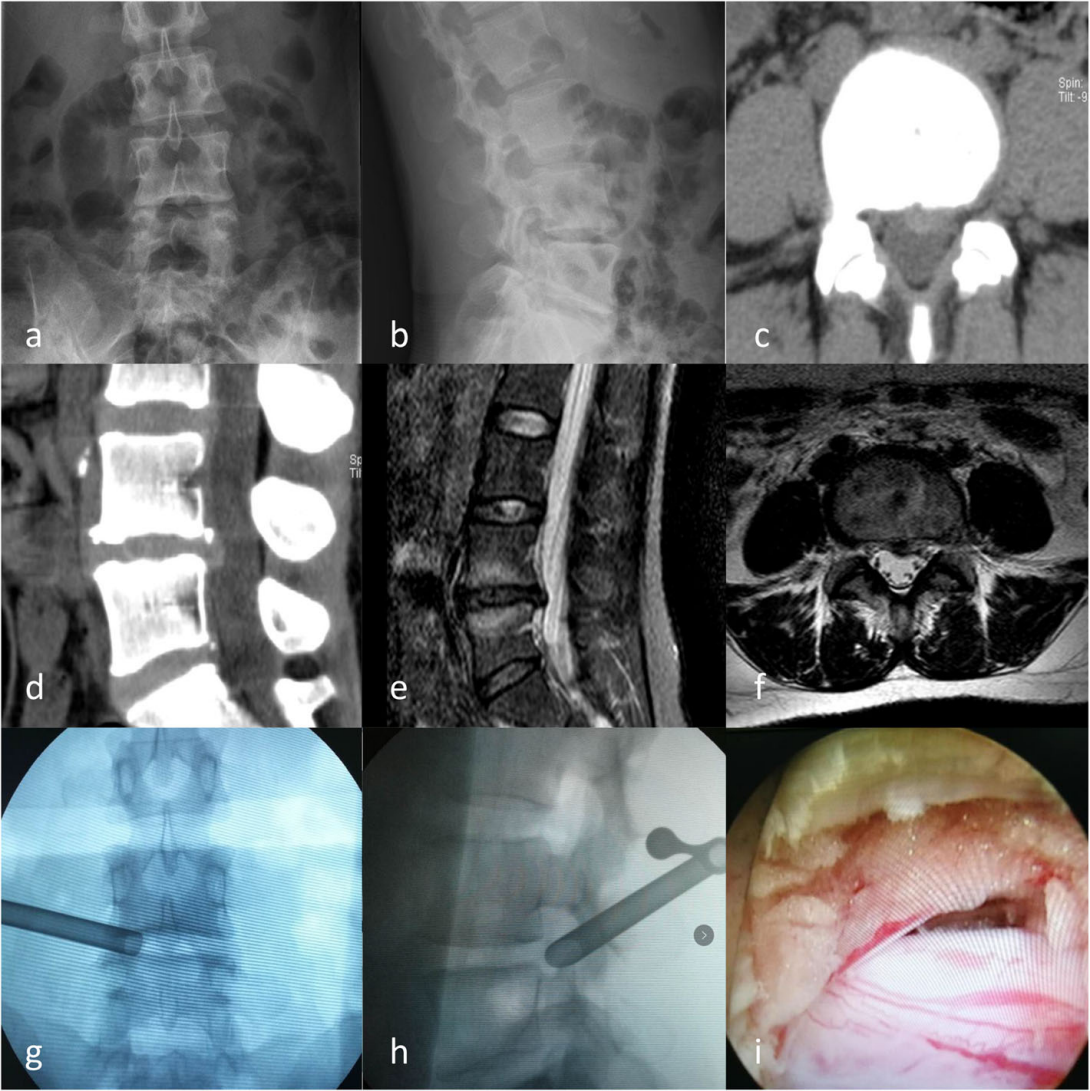
The patient was a 36-year-old female and had a protrusion in the posterior left portion of the L4/5 interverbral discs. **a** X-ray of anterior and posterior lumbar spine before operation. **b** Preoperative lateral X-ray of lumbar spine. **c** In the cross-section, CT revealed a protrusion in the posterior left portion of the L4/5 interverbral discs, which compressed the left L5 nerve root. **d** CT revealed a left L4/5 interverbral disc protrusion in the sagittal section. **e** MRI revealed a left L4/5 interverbral disc protrusion in the sagittal section. **f** In the cross-section, it revealed a protrusion in the posterior left portion of the L4/5 interverbral discs, which compressed the left L5 nerve root. **g** Placement of the working channel during the operation (anteroposterior film). **h** Placement of the working channel during the operation (lateral film). **i** The left L5 nerve root was exposed during the operation

All operations were performed under local anesthesia and monitoring. The patient was placed in a prone position on a special bed for fluoroscopic spine injection with the abdomen suspended. The median line of the spinous process and the contour line of the iliac bone were marked on the surface of the body prior to the operation. A line parallel to the surgical puncture intervertebral disc was determined by fluoroscopy with the guidance of a C-arm X-ray machine. An 8–14-cm line was marked parallel along the posterior median line of the spine on the operative side, and the head was tilted to the side along the horizontal line of the intervertebral space under the guidance of an anteroposterior projection. The intersection between the oblique line through the intervertebral foramen and the parallel line between the intervertebral space was the puncture point. After local infiltration, anesthesia was administered with 1% lidocaine on the skin, and in the superficial fascia at the puncture point, the puncture needle was inserted slowly into the shoulder of the superior articular process of the lower vertebral body under fluoroscopic guidance. Then, local infiltration anesthesia was administered to the articular process joints, the core of the needle was withdrawn, the guide wire was inserted through the puncture needle, an approximately 7 mm incision was made in the skin, and the expansion sleeve and ring saw were inserted step by step along the guide wire with the use of a mirror. Under endoscopy, nucleus pulposus forceps were used to grasp the soft tissue on the surface of the articular process, the bone was exposed, and the articular process bone was rotated with a circular saw. The sawed bone was completely removed with the circular saw and was inserted into a working cannula through the sawed space (Fig. 1 g, h). The yellow ligament on the dorsal side of the nerve root and dural sac was grasped under endoscopy, and dorsal decompression was performed. After decompression, the working cannula was moved to the lateral and ventral sides of the nerve root. A second fluoroscopy showed that the tip of the cannula did not exceed the line of the spinous process and that the lateral position did not exceed the line of the posterior edge of the vertebral body. The protruding nucleus pulposus was removed under endoscopy to expose the nerve root and dural sac (Fig. 1i). In areas with ossification, the nerve root could be treated by a grinding drill and reverse curettage, and the entrance and exit of the nerve root could be loosened throughout until the dural sac pulsated with the patient’s breath and the nerve root relaxed completely without compression. Bipolar radiofrequency ablation was carefully used to stop the bleeding and suture the skin. Anti-infection, dehydration, and detumescence agents were given as appropriate after the operation.

### Grouping

**Fig. 2:**
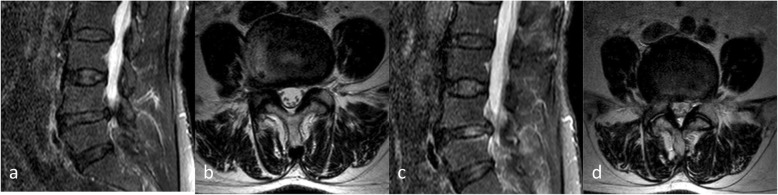
The patient was a 75-year-old female (group E) and had a protrusion in the posterior right portion of the L4/5 interverbral discs. **a** MRI revealed a right L4/5 interverbral disc protrusion in the sagittal section. **b** In the cross-section, it revealed a protrusion in the posterior right portion of the L4/5 interverbral discs, which compressed the right L5 nerve root. **c** Three months after the operation, MRI revealed high signal in the L4/5 vertebral endplate area. **d** In the cross-section, it revealed the high signal intensity in the horizontal nerve root canal of the left L4/5 intervertebral disc

**Fig. 3:**
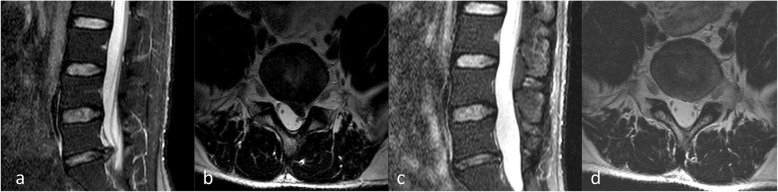
The patient was a 32-year-old male (group L) and had a protrusion in the posterior left portion of the L5/S1 interverbral discs. **a** MRI revealed a left L5/S1 interverbral disc protrusion in the sagittal section. **b** In the cross-section, it revealed a protrusion in the posterior left portion of the L5/S1 interverbral discs, which compressed the left S1 nerve root. **c**, **d** Three months after the operation, in the sagittal section and the transverse section, it revealed no significant protrusion in the L5/S1 interverbral discs, and the nerve roots were not compressed

According to the time to first ambulation after the operation, the patients were divided into three groups: group E (3–24 h), group M (24–72 h), and group L (3–7 days). Many patients who wake up early after PELD will tell us that they feel pain around the wound and that they feel sore in their backs after a long walk. Therefore, we will inform patients that PELD is a minimally invasive operation, and they can become active the next day after surgery. Of course, they can also choose to extend their bed rest as needed, but to no more than 7 days. Finally, the time until getting out of bed was determined by the patient him- or herself. The specific time that the patient got out of bed was recorded (Figs. 2 and 3).

### Evaluation of efficacy

All patients were followed up for at least 3 months. The visual analog scale (VAS) and Oswestry disability index (ODI) scores before the operation, at first ambulation, 1 month after the operation, and 3 months after the operation; recurrence rates; and incidence rates of high MRI signal in the vertebral endplate area were recorded after operation.

### Statistical methods

The data were analyzed by SPSS 23.0 software, and the measurement data were expressed as the mean ± standard. Paired *t* tests were used for intragroup comparisons, variance analyses were used for intergroup comparisons, and chi-square tests were used for counting data. The difference was considered statistically significant at *P* < 0.05.

## Results

### General results

All patients in the three groups were successfully operated on under local anesthesia. The postoperative CT showed that the prominent nucleus pulposus was removed, the intervertebral foramen was decompressed adequately, and the dural sac was not compressed (Fig. 1). None of the patients experienced any serious complications, such as nerve root injury, cauda equina syndrome, intervertebral discitis, hematoma, or wound infection. There were no significant differences in age, sex, preoperative VAS scores of low back pain and leg pain, preoperative ODI, operation time, and bleeding volume among the three groups (Table 1). There were significant differences in hospital stay among the three groups (Table 1), mainly due to the difference in postoperative bed rest time, that is, the time until first walk after operation.

**Table 1:** Patient characteristics

	Group E	Group M	Group L	*P*^▽^
Number	32	30	28	
Age (yr ± SD)	40.81 ± 11.82	39.20 ± 10.97	37.46 ± 10.83	0.518
Gender (male to female)	15:17	16:14	15:13	0.837
Operation time (min)	149.19 ± 25.98	146.83 ± 24.67	147.36 ± 25.56	0.929
Blood loss (ml)	19.50 ± 8.09	18.80 ± 8.87	18.75 ± 6.33	0.908
Before operation lumbar pain VAS	3.81 ± 1.09	3.63 ± 0.93	3.39 ± 0.87	0.255
Before operation leg pain VAS	7.34 ± 1.12	7.17 ± 1.44	7.07 ± 1.56	0.738
Before operation ODI	57.50 ± 7.53	63.04 ± 15.50	58.49 ± 14.20	0.202
The time until first ambulation (h)	21.66 ± 1.64	62.77 ± 14.26	125.93 ± 30.31	0.000
Hospital stay(day)	1.91 ± 0.86	3.63 ± 0.81	6.29 ± 1.34	0.000

^▽^*P* value of the gender is calculated by chi-square test; others are calculated by variance analysis, and *P* value of the hospital stay is calculated by Welch test

*yr* year, *SD* standard deviation

### Comparison of VAS scores

**Fig. 4:**
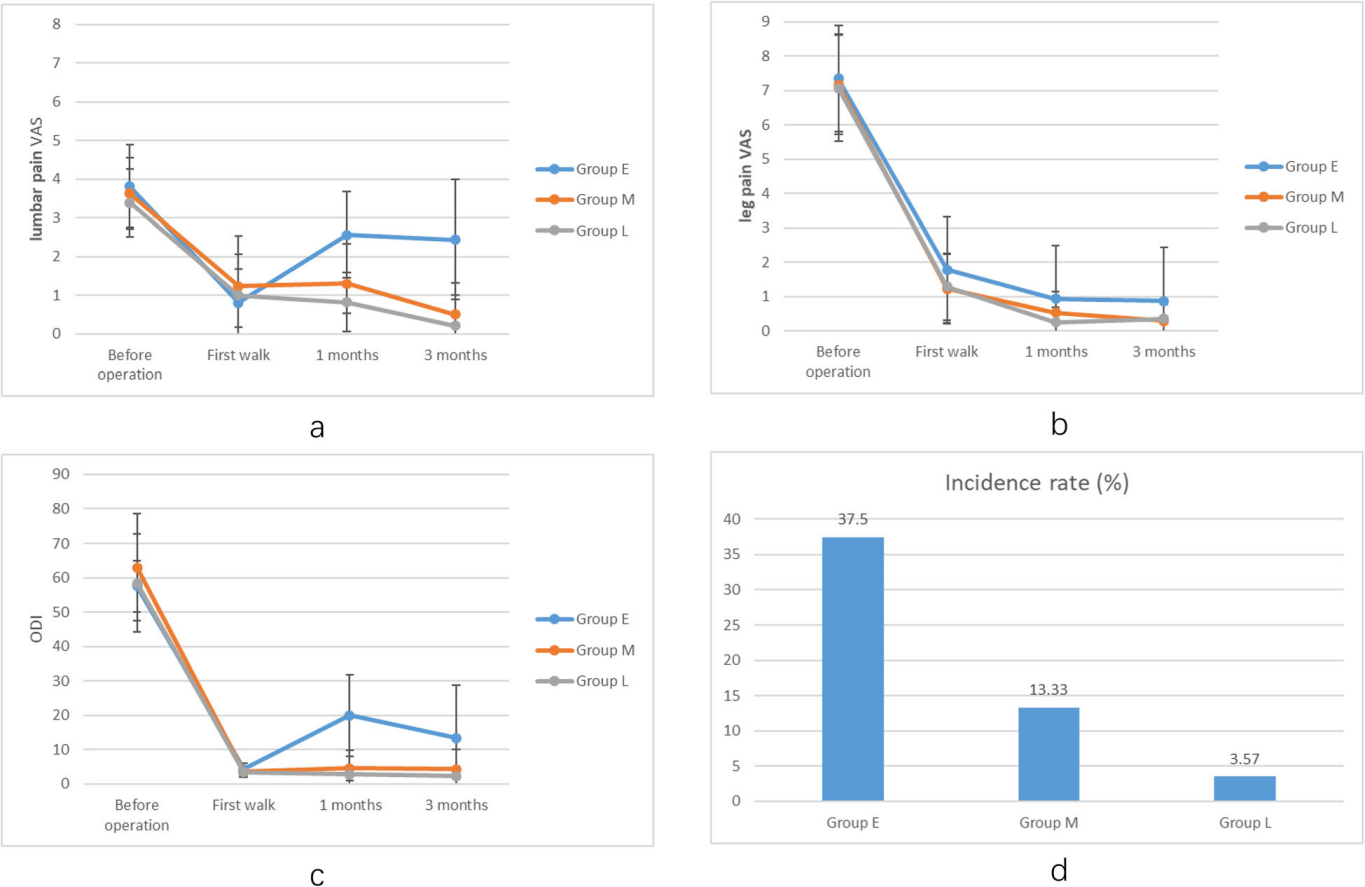
**a**, **b**, **c** Comparison of the lumbar pain and leg pain visual analog scale and ODI by the time in the three groups. **d** Comparisons of the incidence rate of high MRI signal among the three groups

The VAS scores of low back pain and leg pain in the three groups significantly improved at first ambulation after the operation compared with those before the operation (*t* = 21.13, 7.67, 9.43, *P* < 0.05) (Table 2), but there were no significant differences among the three groups (*F* = 1.169, *P* = 0.316) (Table 3). The VAS scores of low back pain in group E at 1 and 3 months after the operation (2.56 ± 1.11, 2.44 ± 1.56) were significantly higher than those in the other two groups (group M 1.30 ± 1.02, 0.50 ± 0.82; group L 0.82 ± 0.77, 0.21 ± 0.79) (*F* = 25.454, *P* < 0.05; *F* = 34.932, *P* < 0.05) (Table 3, Fig. 4a). The VAS scores of leg pain at first ambulation after the operation in group E were significantly higher than those in the other two groups (*F* = 3.600, *P* = 0.031). However, at 1 and 3 months after the operation, there were no significant differences in the VAS scores of leg pain among the three groups (*F* = 2.596, *P* = 0.080; *F* = 2.550, *P* = 0.084) (Table 4, Fig. 4b).

**Table 2:** Comparison of the in the three groups

	Group E	Group M	Group L
Lumbar pain VAS			
Before operation	3.81 ± 1.09	3.63 ± 0.93	3.39 ± 0.88
First walk	0.81 ± 0.86*	1.23 ± 1.30*	1.00 ± 1.05*
	***t*= 21.13**, ***P*< 0.05**	***t*= 7.67**, ***P*< 0.05**	***t*= 9.43**, ***P*< 0.05**
Leg pain VAS			
Before operation	7.34 ± 1.12	7.17 ± 1.44	7.07 ± 1.56
First walk	1.78 ± 0.66*	1.23 ± 1.01*	1.29 ± 0.98*
	***t*= 26.45**, ***P*< 0.05**	***t*= 17.34**, ***P*< 0.05**	***t*= 16.18**, ***P*< 0.05**
ODI			
Before operation	57.50 ± 7.53	63.04 ± 15.50	58.49 ± 14.19
First walk	4.30 ± 1.60*	3.55 ± 1.11*	3.41 ± 1.54*
	***t*= 41.51**, ***P*< 0.05**	***t*= 20.75**, ***P*< 0.05**	***t*= 21.52**, ***P*< 0.05**

Values are mean ± SD

*Statistically significant

**Table 3:** Comparison of the lumbar pain visual analog scale by the time in the three groups

VAS	Group E	Group M	Group L	*F*	*P*
Before operation	3.81 ± 1.09	3.63 ± 0.93	3.39 ± 0.88	1.389	0.255
First walk	0.81 ± 0.86	1.23 ± 1.30	1.00 ± 1.05	1.169	0.316
1 month	2.56 ± 1.11	1.30 ± 1.02	0.82 ± 0.77	25.454	0.000*
3 months	2.44 ± 1.56	0.50 ± 0.82	0.21 ± 0.79	34.932	0.000*

Values are mean ± SD

*Statistically significant

**Table 4:** Comparison of the leg pain visual analog scale by the time in the three groups

VAS	Group E	Group M	Group L	*F*	*P*
Before operation	7.34 ± 1.12	7.17 ± 1.44	7.07 ± 1.56	0.305	0.738
First walk	1.78 ± 0.66	1.23 ± 1.01	1.29 ± 0.98	3.600	0.031
1 month	0.94 ± 1.83	0.53 ± 0.63	0.25 ± 0.44	2.596	0.080
3 months	0.88 ± 1.74	0.30 ± 0.47	0.36 ± 0.49	2.550	0.084

Values are mean ± SD

### Comparison of ODI

The ODI scores of the three groups improved significantly at first ambulation after the operation (Table 2). At first ambulation and at 1 and 3 months after the operation, the ODI scores of group L were significantly better than those of the other two groups (Table 5, Fig. 4c).

**Table 5:** Comparison of the ODI by the time in the three groups

ODI	Group E	Group M	Group L	*F*	*P*
Before operation	57.50 ± 7.53	63.04 ± 15.50	58.49 ± 14.19	1.626	0.203
First walk	4.30 ± 1.60	3.55 ± 1.11	3.41 ± 1.54	3.447	0.036
1 month	19.93 ± 11.79	4.52 ± 5.33	2.85 ± 1.80	45.496	0.000*
3 months	13.47 ± 15.42	4.37 ± 5.64	2.30 ± 0.42	11.359	0.000*

Values are mean ± SD

*Statistically significant

### Comparisons of the incidence rate of high MRI signal in the vertebral endplate area and the recurrence rate

MRI with T2-weighted imaging (T2WI) and fat-suppression sequences displayed that the following proportions of intervertebral discs or endplates had higher signal bands 3 months after the operation than before the operation: 37.50% (group E), 13.33% (group M), and 3.57% (group L). There were significant differences among the three groups (Table 6, Fig. 4d). Because the high signal area occurred in the disc or endplate area and was still present at review 3 months after surgery, these findings were probably due to an inflammatory response, and the possibility of hematomas was small. There were four cases of recurrence in group E, and the recurrence rate was 12.5%. There were no recurrences in the other two groups. The recurrence rate was significantly different among the three groups (Table 7).

**Table 6:** Comparisons of the incidence rate of high MRI signal among three groups

	Group E	Group M	Group L	*P*
Incidence rate (%)	37.50 (12/32)	13.33 (4/30)	3.57 (1/28)	0.002

**Table 7:** Comparison of the recurrence rate in the three groups

	Group E	Group M	Group L	*P*
Recurrence rate (%)	12.5 (4/32)	0 (0/30)	0 (0/28)	0.023

## Discussion

PELD has been used to treat lumbar intervertebral disc herniation for more than 20 years. Compared with traditional open surgery, PELD has the advantages of being minimally invasive, leading to less bleeding and having a faster recovery. These advantages have been confirmed by a large number of clinical cases and are accepted and respected by an increasing number of patients and doctors. However, the problem of recurrence after lumbar intervertebral disc surgery has not been solved for either open surgery or minimally invasive PELD surgery. Postoperative recurrence mainly manifests as recurrent lumbar or leg pain or the simultaneous recurrence of lumbar and leg pain; in most cases, recurrence occurs within 6 months after surgery. As the number of PELD cases is on the rise, increasingly more recurrences have become increasingly more prominent. According to a large number of clinical reports, the recurrence rate of PELD is less than 12.5% [1, 2]. The main factors that influence recurrence are sex, age, body mass index, type and location of disc herniation, choice of surgical approach, operation time, learning curve of the surgeon, etc. The question of whether the time to ambulation will affect recurrence after the operation has not received due attention because many doctors and patients are most concerned about getting the patient out of bed as soon as possible to achieve rapid a recovery. However, regardless of the lumbar vertebral protection method adopted, early ambulation will certainly subject the disc to a load too soon after lumbar surgery, and if the disc suffers surgical trauma, it cannot be fully repaired. Early ambulation is likely one of the factors affecting the recurrence of low back pain or leg pain after PELD, and proper attention should be paid to this issue.

The pathological basis of lumbar disc herniation is the change in intradiscal pressure (IDP), which leads to the rupture of the peripheral fibrous ring, prolapse of the nucleus pulposus, immune expression of inflammatory factors, and eventually the symptoms of low back pain and even radiating pain in the lower limbs with nerve root damage. The internal pressure of the intervertebral disc is mainly affected by the axial load of the spine [3]. In healthy intervertebral discs, the pressure from the nucleus pulposus and the inner annulus is uniform and equivalent to hydrostatic pressure; however, in the outer annulus, the pressure decreases and is directionally dependent. IDP is affected by body posture. The pressure is greatest in front of the flexed disc and in the back of the overextended disc. Pressure maps show that degenerative changes usually include local stress concentrations in the mid-sagittal position. The nucleus pulposus experiences less pressure, and there are many pressure peaks in the annulus fibrosus, especially in the posterior region, which may be related to the tearing and breakage of the annulus fibrosus. According to the “stone in the shoe” principle, these stress concentrations may be the cause of discogenic pain [4]. Several studies have reported using needle-shaped pressure sensors to measure IDP in healthy people during various daily activities [5]. The baseline pressure of the intervertebral disc at rest in the supine position is 0.1–0.2 MPa; this pressure originates from the joint action of the paravertebral muscle load and osmotic pressure in the intervertebral disc. When a person is standing, this pressure changes to 0.5 MPa. In all types of daily activities, the IDP increased significantly and reached 2.3 MPa when the flexion position was loaded. Human-specific stress stimuli during orthostatic activities and in orthostatic positions are also considered to be associated with a high incidence of spinal-related diseases [6].

During various activities of the spine, the tissue of the intervertebral disc expands, and concentric annular fibrous rings are the basis of resistance to tension, pressure, and strain. From the perspective of anatomical factors, there is a clear causal relationship between the loss of the fibrous ring and the occurrence of lumbar and leg pain after lumbar disc herniation. The main nerve supply of the outer fibrous ring consists of small, unmyelinated free nerve endings. After the formation of fissures in the fibrous ring, vascular tissue and nerve endings can penetrate the deep layer of the intervertebral disc and play an important role in causing degenerative disc pain [7].

Regardless of whether lumbar disc herniation is inclusive or noninclusive, the outer fibrous ring is damaged after PELD. If the disc is damaged too early after PELD, it will bear tremendous pressure. However, the healing process of the outer fibrous ring may not be sufficient to effectively reconstruct the outer fibrous ring of degenerative intervertebral discs. In summary, early standing and walking after surgery will cause the damaged intervertebral disc to bear tremendous pressure, leading to local nerve stimulation and increasing the risk for low back pain or radiating pain in the lower extremities. This finding is consistent with the conclusions of this study. The degree of low back pain or lower limb pain in the early stage after waking up from surgery was significantly higher than that in the late stage after waking up (Table 2). According to the results of a meta-analysis, the incidence of early recurrence was nearly twice as high as the late recurrence rate, and patients with early recurrence accounted for the majority of patients with recurrent herniations [8]. Therefore, to determine if there is a correlation among these factors, further research and exploration are still needed.

There are still many controversies about the factors that influence recurrent low back pain or radiating pain in the lower extremities after PELD. Other probable predictive factors include sex, smoking, degenerated disc, modic changes, and the learning curve of the surgeon; the roles of these factors remain controversial. However, it has been reported that age, sex, smoking status, level of herniation, and duration of symptoms are not associated with a high rate of recurrence after partial laminectomy and discectomy [9]. For additional procedures, the pooled result of a subgroup analysis showed that there were no obvious differences between the recurrence rates after PELD with or without foraminoplasty [8].

Older discs are often degenerative, and the remaining fragment of the nucleus pulposus is susceptible to prolapse in response to a mechanical overload; this event can be caused by an annular incision during surgery [10]. Likewise, excess weight due to cyclical increase in the IDP could lead to high shear strains in the posterolateral part of the annulus fibrosus, which would result in disc herniation [11, 12]. This study also found that early ambulation after PELD may lead to the re-protrusion of the nucleus pulposus and low back pain or radiating pain in the lower extremities because the damaged fibrous rings are not repaired in time. Because of the anatomical relationship between the lumbar intervertebral disc and the nerve root, the clinical manifestations of the central prominence of the nucleus pulposus are simple low back pain. When the nucleus pulposus is protruded to one side, the nerve root is compressed, which is accompanied by radiating pain in the lower extremities and other clinical manifestations. One large sample-based study reported a recurrence rate of 4.7% for central disc herniation, which was significantly higher than that of paramedian herniations (2.7%, *P* = 0.008) [13]. This finding is consistent with the results of the current study. The most common early complication after PELD is low back pain. The degree of low back pain in the early wake-up group was significantly higher than that in the other two groups within 3 months after the operation, but there was no significant difference in the degree of leg pain between the latter two groups.

Some studies have speculated that the unique anatomical environmental factors of the upper lumbar level, such as the small spinal canal, the larger dural sac, and the conus medullaris in the dural sac, and the inherent technical difficulty of walking might be the main reasons for the high recurrence rate [14]. However, in the current study, no relationships between low back pain after PELD and operative segment were observed or compared, and this is the focus of the next research study.

The choice of the PELD working channel may be one of the influencing factors of low back pain after the operation. For the central prominence of the nucleus pulposus, the working channel is placed inside the nucleus pulposus with a very steep trajectory angle. As a result, the ruptured intervertebral disc is not easily accessible, and the choice of working channel position may be the main cause of the high recurrence rate [15].

## Conclusion

Many factors influence recurrence after PELD, but the time to first ambulation after the operation is certainly one of the factors. Few studies have discussed this aspect. In comparison, the current study incorporates fewer samples, more influencing factors, and many shortcomings. More detailed and precise research is still needed to confirm this hypothesis.

## Data Availability

Not applicable.

## Abbreviations

CT: Computed tomography
IDP: Intradiscal pressure
MRI: Magnetic resonance imaging
ODI: Oswestry disability index
PELD: Percutaneous endoscopic lumbar discectomy
VAS: Visual analog scale
